# Are workplace health promotion programmes effective for all socioeconomic groups? A systematic review

**DOI:** 10.1136/oemed-2019-106311

**Published:** 2020-03-26

**Authors:** David van de Ven, Suzan J W Robroek, Alex Burdorf

**Affiliations:** Department of Public Health, Erasmus MC, University Medical Center Rotterdam, Rotterdam, The Netherlands

**Keywords:** occupational health practice, health promotion, public health

## Abstract

Decreasing socioeconomic health inequalities is considered an important policy priority in many countries. Workplace health promotion programmes (WHPPs) have shown modest improvements in health behaviour. This systematic review aims to determine the presence and magnitude of socioeconomic differences in effectiveness and the influence of programme characteristics on differential effectiveness of WHPPs. Three electronic databases were searched for systematic reviews published from 2013 onwards and for original studies published from 2015 onwards. We synthesised the reported socioeconomic differences in effectiveness of WHPPs on health behaviours, and calculated effectiveness ratios by dividing the programme effects in the lowest socioeconomic group by the programme effects in the highest socioeconomic group. Thirteen studies with 75 comparisons provided information on the effectiveness of WHPPs across socioeconomic groups. Ten studies with 54 comparisons reported equal effectiveness and one study with 3 comparisons reported higher effectiveness for lower socioeconomic groups. Quantitative information on programme effects was available for six studies with 18 comparisons, of which 13 comparisons showed equal effectiveness and 5 comparisons showed significantly higher effect sizes among workers in low socioeconomic position. The differential effectiveness of WHPPs did not vary across programme characteristics. In this study no indications are found that WHPPs increase socioeconomic inequalities in health behaviour. The limited quantitative information available suggests that WHPPs may contribute to reducing socioeconomic inequalities. Better insight is needed on socioeconomic differences in effectiveness of WHPPs to develop strategies to decrease socioeconomic inequalities in health in the workforce.

**Key messages**

**What is already known about this subject?**

- Workplace health promotion programmes (WHPPs) result in modest reductions in body weight and modest increases in healthy nutrition and physical activity of workers.
- Evidence on the differential effectiveness of WHPPs across socioeconomic groups and the influence of programme characteristics on the differential effectiveness is lacking.

**What are the new findings?**

- The majority of the studies reported an equal effectiveness of WHPPs across socioeconomic groups without providing quantitative information on programme effects.
- Some studies reported higher effectiveness among those in lower socioeconomic position.
- Quantitative information suggests that most WHPPs are equally effective across socioeconomic groups, and some studies showed higher effectiveness among workers in low socioeconomic position.

**How might this impact on policy or clinical practice in the foreseeable future?**

- WHPPs can be an effective strategy to decrease socioeconomic inequalities in health in the workforce.

## Introduction

It is well known that unhealthy behaviours such as smoking, alcohol intake, unhealthy diet and lack of physical activity have adverse effects on workers’ health.^1^ These unhealthy behaviours are also related to productivity loss at work and higher sickness absence.^2–4^ Unhealthy behaviours are more prevalent among lower socioeconomic groups and contribute to socioeconomic inequalities in health.^5 6^ Numerous workplace health promotion programmes (WHPPs) have been developed to improve health and health behaviours of workers. However, more insight is needed concerning the extent to which these programmes are effective for different socioeconomic groups and which programme characteristics contribute to decreasing socioeconomic health inequalities. This may provide input for policy makers on which programmes should be implemented at a large scale to promote the health of workers in lower socioeconomic position, thereby contributing to the priority in various European regions and countries to improve health equity.^7^

Ever since the 1950s the workplace has received international recognition as an important setting for health promotion.^8^ The workplace offers certain advantages in that a large proportion of individuals can be reached and that multiple levels of influence (individual, interpersonal and organisational) can be addressed.^9^ In recent years, several systematic reviews have reported on the effectiveness of WHPPs. They have shown reductions in body weight in the short term,^10–12^ increases in mean daily steps,^11^ and increases in consumption of fruits and vegetables.^13 14^ However, because these reviews only investigated the overall effects of WHPPs, they lack information on differential effects across socioeconomic groups.

A few meta-analyses and systematic reviews have compared the effects of WHPPs provided to workers in lower socioeconomic positions with programmes universally provided or those targeted to workers in higher socioeconomic positions. A meta-analysis from 2013 concludes that studies with predominantly white-collar workers reported higher effectiveness of WHPPs (effect size=0.33).^15^ In contrast, Cairns *et al* 16 have shown that, while most studies found no effects on body mass index (BMI) or body weight, two intensive multicomponent interventions targeted towards workers in lower socioeconomic positions showed reductions in body weight of 2 kg. Furthermore, a meta-analysis from 2014 found overall mean reductions in BMI (effect size by Hedges G=−0.155) and improvements in fruit and vegetable consumption (Hedges G=0.116), but no differences between interventions conducted among workers in lower socioeconomic positions and interventions targeted towards higher socioeconomic groups.^17^ These studies did not compare differential effectiveness within a specific WHPP, but compared WHPPs targeted to workers in lower socioeconomic positions with universally provided WHPPs or those targeted to workers in higher socioeconomic position. To our knowledge, only one systematic review performed subgroup analyses to analyse differences in effectiveness of Dutch workplace obesity prevention interventions across socioeconomic groups.^18^ This systematic review found that two of the six Dutch obesity prevention interventions in the workplace setting were more effective for workers in higher socioeconomic groups, compared with workers in lower socioeconomic positions. For the four remaining workplace interventions, no differential effect was observed.

From these studies the extent to which the effects of WHPPs differ across socioeconomic groups and which programmes decrease socioeconomic inequalities in health behaviour remain inconclusive. Our systematic review aims to (1) determine the presence and magnitude of differences in the effectiveness of WHPPs across socioeconomic groups, and (2) evaluate the influence of programme characteristics on the differential effectiveness of WHPPs.

## Methods

### Search strategy and selection criteria

We conducted a two-tier search by selecting relevant studies through reviews. On 9 July 2018, Embase, Medline Ovid, and Cochrane Database of Systematic Reviews (DSR) and Database of Abstracts of Reviews of Effects (DARE) were searched for reviews published since 2013 using terms related to (1) workplace, (2) lifestyle (smoking, nutrition, alcohol intake, physical activity, body weight or BMI) and (3) study design with a control group. On 13 November 2018, an additional search was performed of original studies published from 2015 onwards, in order to also include the most recent original studies on the effectiveness of WHPPs that have not been included in existing reviews. An overview of the search strategy for each electronic database is presented in online supplementary appendix A. We chose to search for studies through reviews because of the high volume of studies published on WHPPs. We assume that older studies are included in the reviews published since 2013.

10.1136/oemed-2019-106311.supp1 Supplementary data

In order to be eligible, reviews and individual studies had to meet the following inclusion criteria: (1) the health promotion programmes are carried out in, or stimulated through, the workplace; (2) evaluation of the effectiveness of WHPPs on smoking cessation, healthy nutrition, reduction in alcohol intake, increase in physical activity, and reduction in body weight or BMI; (3) effectiveness of these programmes was determined with at least one before and after measurement with a control group; and (4) evaluation of differences in effectiveness between socioeconomic groups.

### Data extraction and quality assessment

The studies were first screened on title and abstract. This was independently done by two researchers (DvdV and SJWR). The full-text papers were investigated by one researcher (DvdV), and the results of the selection of relevant studies were checked by a second researcher (SJWR). Using a data extraction form, one researcher (DvdV) summarised information on the population (eg, distribution of socioeconomic groups, type of companies), study design (eg, type of design, randomisation procedure, follow-up period), intervention (eg, type of intervention, frequency of contact) and analysis (eg, outcome measures, differential effectiveness by socioeconomic group). The second researcher (SJWR) checked the data extraction.

Appraisal of the methodological quality of the studies was independently done by two researchers (DvdV and SJWR) using a nine-item checklist (online supplementary appendix B). This checklist is based on The Cochrane Collaboration’s tool for assessing risk of bias,^19^ and was used earlier in a meta-analysis on the effectiveness of WHPPs.^15^ Studies received a score of 0 when the quality criterion was not met or no sufficient information was provided, and a score of 1 when the quality criterion was met. When studies investigated multiple outcomes, they could receive a score of 0.5 on criteria related to the similarity of groups and the use of objective measures when they were met for a part of the total number of outcomes. The scores for each study were added up and were divided into excellent (8–9), good (4.5–7.5), fair (3–4) and poor (0–2.5) quality. Discrepancies between the two researchers were resolved through discussion, and if consensus was not reached a third researcher (AB) was involved.

### Data analysis

All included studies were synthesised. First, the study findings on the overall effectiveness of WHPPs were summarised, whereby multiple comparisons on different health behaviours per study were possible. Second, the socioeconomic differences in the effectiveness of WHPPs were evaluated. A distinction was made between studies that reported on the presence or absence of differential effectiveness without providing further quantitative details (qualitative information) and studies with quantitative information that allowed calculation of the effectiveness ratio as the ratio of programme effects among workers in low socioeconomic position over the programme effects among workers in high socioeconomic position. The effectiveness ratios were calculated by dividing the programme effects in the lowest socioeconomic group by the programme effects in the highest socioeconomic group. Decisions on the significance of differential effects were based on 95% CI or p values (p<0.05) as presented in the studies. In addition, for each outcome a pooled effect estimate was calculated as a weighted average taking into account the study sample size. Third, we investigated which types of programmes (programme components, universal vs selective, involvement of workers in lower socioeconomic groups in programme development, primarily designed for workers in lower socioeconomic groups) were more effective for workers in lower socioeconomic positions.

## Results

### Selection of studies

**Figure 1:**
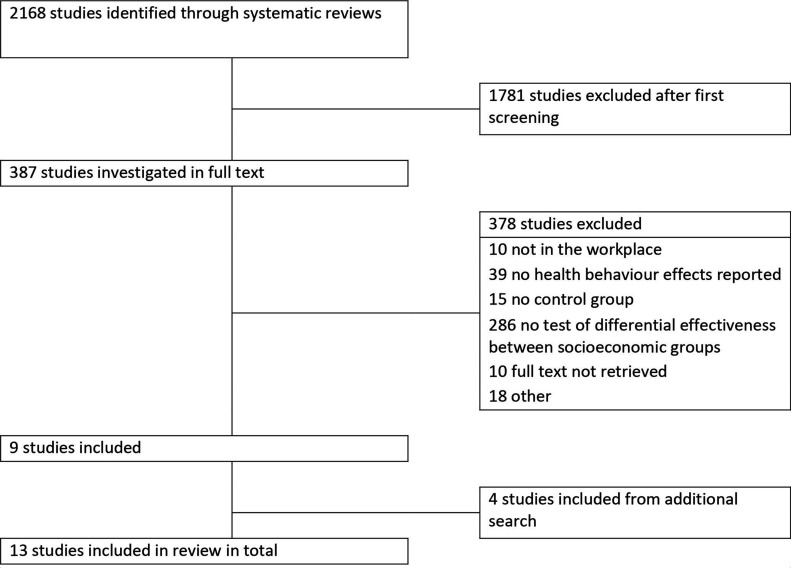
Flow chart of literature search.

From systematic reviews published from 2013 onwards, 2168 original studies were identified, of which 9 studies ultimately fulfilled our inclusion criteria (figure 1). Most studies were excluded because they did not evaluate differences in effectiveness across socioeconomic groups (n=286). The additional search of studies published from 2015 onwards yielded 3831 titles, of which 4 studies were eligible for inclusion. Thus, a total of 13 studies were included in this systematic review. The complete flow charts for both the search through reviews as well as the additional search for original studies are presented in online supplementary appendix C.

### Study characteristics

The characteristics of the included studies can be seen in table 1. The 13 included studies reported the differential effectiveness of interventions by socioeconomic group on physical activity (n=7),^20–26^ smoking (n=6),^20 26–30^ nutrition (n=6),^21–23 26–28^ and BMI or body weight (n=5).^22–24 31 32^ None of the studies investigated alcohol intake as outcome. Studies mostly consisted of cluster-randomised controlled trials (n=6)^21 23 27–30^ or randomised controlled trials (n=4).^22 24 25 32^

**Table 1:** Characteristics of included studies

Study	Design	Intervention type	Outcome measure	Total effect of WHPPs
Bergström *et al* 20	CT	I	Proportion of smokers.	T10 (3 years): change in proportion intervention groups-reference group: −0.09 to −0.92 (3 of the 4 companies, p<0.05).
			Proportion of workers performing regular exercise (≥2 times/week).	T10 (3 years): change in proportion intervention groups–reference group: −0.10 to 1.09 (none of the 4 companies, p<0.05).
Sorensen *et al* 21	Cluster-RCT	II and III	Proportion of workers eating ≥5 servings of fruits and vegetables/day.	T1 (18 months): intervention: +5.4%, control: +1.7% (p=0.41).
			Proportion of workers eating ≤3 servings of red meat/week.	T1 (18 months): intervention: +4.1%, control: +3.0% (p=0.72).
			Proportion of workers performing ≥2.5 hours of PA/week.	T1 (18 months): intervention: +5.4%, control: −0.9% (p=0.23).
Sorensen *et al* 27	Cluster-RCT	II and III	Mean percentage of kilocalories from fat.	T1 (2 years): intervention: −3.36%, control: −1.55% (p=0.01).
			Mean grams of fibre per 1000 kilocalories.	T1 (2 years): intervention: +8%, control: +5% (p=0.08).
			Mean servings of fruits and vegetables.	T1 (2 years): intervention: +9.4%, control: +3.9% (p=0.04).
			6-month smoking abstinence.	T1 (2 years): intervention: 15%, control: 9% (p=0.123).
Sorensen *et al* 28	Cluster-RCT	I and II	6-month smoking abstinence.	T1 (24 months): OR=1.57 (p=0.17).
			Mean servings of fruits and vegetables.	T1 (24 months): intervention: −0.10, control: +0.05 (p=0.24).
Cook *et al* 22	RCT	II	The frequency with which respondents exercise control over their eating during the past 30 days.	T1 (3 months): difference intervention − control: 0.07 (95% CI −0.01 to 0.15, p=0.08).
			Body mass index (kg/m^2^).	T1 (3 months): difference intervention − control: 0.07 (95% CI −0.28 to 0.41, p=0 .70).
			Frequency of strenuous exercise per week.	T1 (3 months): difference intervention − control: −0.11 (95% CI −0.52 to 0.31, p=0.61).
			Frequency of moderate exercise per week.	T1 (3 months): difference intervention − control: 0.47 (95% CI −0.01 to 0.96, p=0.06).
			Frequency of mild exercise per week.	T1 (3 months): difference intervention − control: 1.03 (95% CI 0.26 to 1.81, p=0.01).
			Frequency of overall exercise per week (sum of previous 3).	T1 (3 months): difference intervention − control: 4.98 (95% CI −0.66 to 10.62, p=0.08).
			Frequency of activity long enough to work up a sweat.	T1 (3 months): difference intervention − control: 0.08 (95% CI −0.08 to 0.23, p=0.33).
Robroek *et al* 23	Cluster-RCT	II	Proportion of workers performing sufficient moderate and vigorous intensity (30 min a day or more).	T1 (12 months): OR=1.07 (95% CI 0.73 to 1.55). T2 (24 months): OR=1.01 (95% CI 0.67 to 1.52).
			Proportion of workers performing sufficient vigorous intensity (at least 3 days a week 20 min or more).	T1 (12 months): OR=1.04 (95% CI 0.72 to 1.52). T2 (24 months): OR=0.67 (95% CI 0.44 to 1.03).
			Proportion of workers meeting guidelines of fruit intake (200 g or more a day).	T1 (12 months): OR=1.18 (95% CI 0.82 to 1.72). T2 (24 months): OR=1.22 (95% CI 0.79 to 1.87).
			Proportion of workers meeting guidelines of vegetable intake (200 g or more a day).	T1 (12 months): OR=0.96 (95% CI 0.68 to 1.37). T2 (24 months): OR=0.75 (95% CI 0.51 to 1.12).
			Proportion of workers with obesity (≥30 kg/m^2^).	T1 (12 months): OR=1.56 (95% CI 0.51 to 4.79). T2 (24 months): OR=1.57 (95% CI 0.52 to 4.76).
Slootmaker *et al* 24	RCT	II	Median minutes per week performing light-intensity PA.	T1 (3 months): difference=−129 (95% CI −337 to 79). T2 (8 months): difference=−2.0 (95% CI −210 to 206).
			Median minutes per week spent on moderate-intensity PA.	T1 (3 months): difference=−13.0 (95% CI −89 to 63). T2 (8 months): difference=103 (95% CI −42 to 248).
			Median minutes per week spent on vigorous-intensity PA.	T1 (3 months): difference=−6 (95% CI −75 to 62). T2 (8 months): difference=−28 (95% CI −110 to 54).
			Median minutes per week spent on moderate-intensity to vigorous-intensity PA.	T1 (3 months): difference=−23 (95% CI −121 to 76). T2 (8 months): difference=74 (95% CI −119 to 267).
			Median minutes per week spent sedentary.	T1 (3 months): difference=10 (95% CI −435 to 455). T2 (8 months): difference=−267 (95% CI −803 to 268).
			Body weight (kg).	T1 (3 months): difference=−0.36 (95% CI −1.23 to 0.49).
Scoggins *et al* 31	Prospective cohort	I, II and III	Body mass index (kg/m^2^).	T1 (1 year): difference intervention − control: −1.10% (p=0.01).
Reijonsaari *et al* 25	RCT	I	Mean MET minutes per week.	T1 (6 months): difference intervention − control: −365 MET min/week (95% CI −733 to 3). T2 (12 months): difference intervention − control: −207 MET min/week (95% CI −531 to 116).
Bhiri *et al* 26	CT	II and III	Proportion of workers consuming five servings of fruits and vegetables or more.	T1 (3 years): ratio intervention − control: 1.14 (95% CI 0.88 to 1.48).
			Proportion of people performing at least 150 min of moderate-intensity aerobic PA throughout the week or at least 75 min of vigorous-intensity aerobic PA throughout the week.	T1 (3 years): ratio intervention − control: 0.93 (95% CI 0.71 to 1.22).
			Proportion of smokers.	T1 (3 years): ratio intervention − control: 0.98 (95% CI 0.75 to 1.29).
Carpenter *et al* 32	RCT	I and II	Body weight (% weight loss).	T1 (6 months): Hedges G=−0.15 (95% CI −0.64 to 0.35).
van den Brand *et al* 29	Cluster-RCT	I and V	Biochemically validated smoking abstinence after programme completion.	T1 (after programme completion): OR=1.77 (95% CI 1.00 to 3.12).
			Biochemically validated smoking abstinence after 3 months.	T2 (3 months): OR=1.55 (95% CI 1.07 to 2.24).
			Biochemically validated smoking abstinence after 6 months.	T3 (6 months): OR=2.39 (95% CI 1.62 to 3.52).
			Biochemically validated smoking abstinence after 12 months.	T4 (12 months): OR=1.93 (95% CI 1.31 to 2.85).
Sorensen *et al* 30	Cluster-RCT	I, II and III	6-month smoking abstinence.	T1 (18 months): OR=1.81 (95% CI 0.85 to 3.89).
			30-day smoking abstinence.	T1 (18 months): OR=1.70 (95% CI 0.87 to 3.32).

CT, controlled trial; I, direct coaching; II, educational; III, environmental; MET, metabolic equivalent; PA, physical activity; RCT, randomised controlled trial; V, financial incentive; WHPP, workplace health promotion programme.

The follow-up period varied between 3 months and 3 years from baseline until the last follow-up measurement, and the median size of the study population was 1740, ranging from 75 to 19 559 (online supplementary appendix D). The study population of six studies consisted mainly of workers in lower socioeconomic positions,^20 21 25 26 28 30^ and in seven studies workers with higher socioeconomic positions were over-represented.^22–24 27 29 31 32^ Socioeconomic position was defined based on occupational class (n=7),^20 21 25–28 30^ educational level (n=8)^22–24 26 27 29 31 32^ and household income (n=3).^21 22 29^

For the majority of the studies the methodological quality was rated as good (n=10). One study was rated as excellent, one study as fair and one study was of poor quality (online supplementary appendix E).

### General effect of interventions on health behaviour

Five^20 22 27 29 31^ of the 13 included studies showed statistically significant effectiveness of interventions on health behaviour, that is, smoking cessation (n=2),^20 29^ healthy nutrition (n=1),^27^ increased physical activity (n=1)^22^ and decreased BMI (n=1)^31^ (table 1). In four studies relatively larger improvements in health behaviour were found in the intervention group compared with the control group, without reaching statistical significance.^21 26–28^ These improvements were found for measures of nutrition,^21 26 27^ physical activity^27^ and smoking abstinence.^27 28^ In four studies larger, although non-significant, improvements in health behaviour were found in the control group than in the intervention group.^22 24 26 28^ These effects were found for measures of healthy nutrition^28^ and physical activity.^22 24 26^

### Differences in effectiveness between socioeconomic groups

Of the 13 studies with 75 comparisons of differential effectiveness of WHPPs across socioeconomic groups, 10 studies with 57 comparisons reported in qualitative terms on differential programme effectiveness. Table 2 shows that 10 studies (54 comparisons) reported equal effectiveness of WHPPs across socioeconomic groups and 1 study (3 comparisons) reported a higher programme effectiveness for those in lower socioeconomic position.

**Table 2:** Number of studies and comparisons reporting on the presence or absence of differential effectiveness and effectiveness ratios for each outcome

		Less effective for lower SEP	Equally effective	More effective for lower SEP
Physical activity (n=7, k=32)	Qualitative information		(n=6, k=27)^20–25^	(n=1, k=1)^24^
	Quantitative information		(n=2, k=4), ratio 0.65:2.19^21 26^	n/a
Smoking cessation (n=6, k=16)	Qualitative information		(n=2, k=9)^20 29^	n/a
	Quantitative information		(n=4, k=6), ratio 0.74:3.04^26–28 30^	(n=1, k=1), ratio 3.36^30^
Nutrition (n=6, k=19)	Qualitative information		(n=5, k=13)^21–23 27 28^	n/a
	Quantitative information		(n=1, k=3), ratio 0.54:0.88^26^	(n=2, k=3), ratio 2.18:3.35^21 27^
BMI/body weight (n=5, k=8)	Qualitative information		(n=3, k=5)^22 23 32^	(n=1, k=2)^24^
	Quantitative information		n/a	(n=1, k=1), ratio 1.65^31^
Total	Qualitative information		(n=10, k=54)^20–25 27–29 32^	(n=1, k=3)^24^
	Quantitative information		(n=5, k=13), ratio 0.54:3.04^21 26–28 30^	(n=4, k=5), ratio 1.65:3.36^21 27 30 31^

The sum of studies is higher than 13 because some studies test multiple comparisons of differential effectiveness with different results.

BMI, body mass index; k, number of comparisons; n, number of studies; n/a, not available; SEP, socioeconomic position.

Effectiveness ratios could be calculated in six studies for 18 comparisons. Thirteen comparisons across five studies showed no statistically significant difference in effectiveness, with effectiveness ratios ranging from 0.54 to 3.04 (median=0.93). Five comparisons in four good-quality studies showed significantly higher effect sizes among workers in low socioeconomic positions than workers in high socioeconomic positions, with effectiveness ratios between 1.65 and 3.36 (median=2.80). These differential effects in favour of workers in low socioeconomic position were mostly found for measures of healthy nutrition.

**Figure 2:**
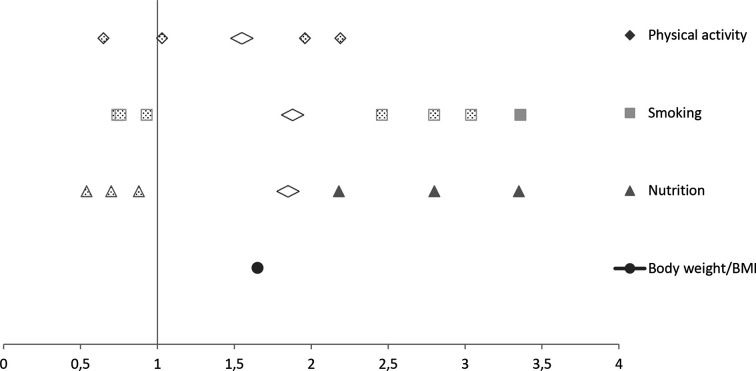
Effectiveness ratios for programme effectiveness among workers in low socioeconomic position compared with those in higher socioeconomic position by outcome. The filled markers refer to significant differential effects of workplace health promotion programmes, and the dotted markers indicate non-significant effects. BMI, body mass index.

Figure 2 shows that effectiveness ratios for increasing physical activity, smoking cessation and healthy nutrition were in favour of both workers in low (effectiveness ratio above 1) and high (effectiveness ratio below 1) socioeconomic positions. The effectiveness ratios were most often in favour of workers in low socioeconomic position for smoking, with a pooled effectiveness ratio of 1.88 (range 0.74–3.36). For nutrition the pooled effectiveness ratio was 1.85 (range 0.54–3.35) and for physical activity 1.55 (range 0.65–2.19).

### Characteristics of WHPPs

Table 3 shows that most of the included WHPPs provided health education (77%), were aimed at universal prevention (77%), did not involve workers in low socioeconomic position in programme development (62%), and were not specifically designed for this group of workers (69%) (in online supplementary appendix F, the extended table on differential effectiveness of WHPPs by programme characteristics is presented).

**Table 3:** Number of studies and comparisons with equal effectiveness across socioeconomic groups or higher effectiveness among workers in lower socioeconomic position by programme characteristics

	Equally effective	More effective for lower SEP
Programme component		
Direct coaching (n=7, k=15)	(n=6, k=13)^20 25 28–30 32^	(n=2, k=2)^30 31^
Education (n=10, k=64)	(n=9, k=56)^21–24 26–28 30 32^	(n=5, k=8)^21 24 27 30 31^
Environmental (n=5, k=25)	(n=4, k=20)^21 26 27 30^	(n=4, k=5)^21 27 30 31^
Financial incentive (n=1, k=1)	(n=1, k=1)^29^	
Population of workers		
Selective (n=3, k=21)	(n=3, k=18)^24 29 32^	(n=1, k=3)^24^
Universal (n=10, k=54)	(n=9, k=49)^20–23 25–28 30^	(n=4, k=5)^21 27 30 31^
Involvement of workers in low socioeconomic position		
Yes (n=5, k=19)	(n=5, k=15)^20 21 27 28 30^	(n=3, k=4)^21 27 30^
No (n=8, k=56)	(n=7, k=52)^22–26 29 32^	(n=2, k=4)^24 31^
Designed for workers in low socioeconomic position		
Yes (n=4, k=17)	(n=4, k=13)^21 27 28 30^	(n=3, k=4)^21 27 30^
No (n=9, k=58)	(n=8, k=54)^20 22–26 29 32^	(n=2, k=4)^24 31^

None of the included studies reported or showed lower programme effectiveness for workers in low socioeconomic position.

The sum of studies is higher than 13 because some studies test multiple comparisons of differential effectiveness with different results.

The number of studies and comparisons in this table reflect both qualitative and quantitative information on differential effectiveness.

k, number of comparisons; n, number of studies; SEP, socioeconomic position.

Of the five studies with higher programme effectiveness for workers in lower socioeconomic position, all consisted of health education interventions, in most cases (four studies) combined with environmental changes (eg, tobacco control policies, increased availability of healthy food), and two provided direct coaching in addition to education and environmental changes. Three of the five studies which were more effective among lower workers in lower socioeconomic position were primarily designed for this group of workers and involved workers in lower socioeconomic groups in the development of the programme.

The characteristics of the WHPPs that were equally effective did not differ statistically significantly from the WHPPs that were more effective for workers in lower socioeconomic position.

## Discussion

### Summary of findings

Thirteen studies (75 comparisons) provided information on programme effectiveness across socioeconomic groups. Ten studies (57 comparisons) reported in qualitative terms on differential programme effectiveness, of which the majority (10 studies with 54 comparisons) reported equal effectiveness across socioeconomic groups, none reported lower effectiveness for workers in low socioeconomic position, and one study (three comparisons) reported higher effectiveness among lower socioeconomic groups. Six studies with 18 comparisons provided quantitative information, of which 13 comparisons showed equal effect sizes and 5 comparisons showed higher programme effects for those in lower socioeconomic position. Pooled effectiveness ratios showed that for each outcome the programmes were more effective for workers in low socioeconomic position. Because of negligible differences in intervention characteristics, there is no clear insight on which types of programmes were most effective for workers in low socioeconomic position.

### Do WHPPs increase socioeconomic inequalities in health behaviour?

According to the ‘inverse prevention law’, public health interventions may increase socioeconomic inequalities in health, because advantaged groups are better able to benefit from these interventions.^33 34^ However, since we did not find lower programme effectiveness for workers in lower socioeconomic position, this systematic review does not support this hypothesis for health promotion programmes in the workforce. Health promotion programmes provided through the workplace may even make a modest contribution to reducing socioeconomic inequalities in health behaviour, as some studies showed higher programme effectiveness among workers in low socioeconomic position.

The presence of higher programme effectiveness for workers in low socioeconomic position in some studies could partially be explained by the relatively high participation of this group of workers in the programmes offered. In two of the five studies with larger effects among workers in lower socioeconomic position, those in lower socioeconomic groups were over-represented compared with workers in higher socioeconomic position.^21 30^ Of the five studies that were more effective among workers in lower socioeconomic position, three were specifically designed for this group of workers by involving them in the development and implementation of the programme.^21 27 30^ However, more studies are needed with sufficient power to test whether these factors are crucial for a higher effectiveness among workers in lower socioeconomic position. Another explanation for the higher programme effectiveness for those in lower socioeconomic position in some studies could be the higher prevalence of unhealthy behaviour for this group of workers at baseline. This would imply more room for improvement for these workers. Unfortunately, we could not test this with the available data in the included studies.

Only a minority of the studies on effectiveness of WHPPs presented quantitative information on differential effectiveness across socioeconomic groups. In these studies socioeconomic subgroups were often small, as these studies were not designed to study differential effects across socioeconomic positions. In studies failing to detect statistical differences in effectiveness between socioeconomic groups up to three times, higher effectiveness was found for workers in low socioeconomic position. The lack of statistically significant differences across socioeconomic groups might therefore be explained by a lack of power.

Despite evidence of some WHPPs being more effective for workers in low socioeconomic position, most programmes were equally effective for lower and higher socioeconomic groups. In line with these results, Magnée *et al* 18 found that most studies (four of the six) showed no differential effects of workplace interventions. Cairns *et al* 16 found that most interventions, regardless of whether they are provided to lower socioeconomic groups or targeted to workers in higher socioeconomic positions, showed no effect on BMI or body weight. In addition, Montano *et al* 17 found no differences between interventions conducted among workers in lower socioeconomic positions and interventions targeted towards higher socioeconomic groups in their meta-analysis. However, the latter two studies did not focus on differential effectiveness of the same interventions across socioeconomic groups, as was done in our review and in the review on Dutch obesity interventions.^18^

In contrast to our review, Magnée *et al* 18 found some evidence for interventions increasing socioeconomic inequalities. They showed that two interventions were more effective for workers in higher socioeconomic groups compared with those in lower socioeconomic position. The authors explained that this differential effect could be the result of the low proportion of participants in lower socioeconomic groups in these studies (on average 36%). Rongen *et al* 15 provided support for this argument in their meta-analysis by showing that interventions with higher compositions of white-collar workers (≥67%) were more effective on various health-related and work-related outcomes. In several studies included in our review, there was an over-representation of workers in lower socioeconomic position (on average 77%)^20 21 25 26 28 30^ and a low number of workers in higher socioeconomic groups in the final analysis.^21 27 28 30^

### Which WHPPs are more effective for workers in low socioeconomic position?

Because of the low number of studies with information on differential effectiveness in WHPPs, the analyses on the influence of programme characteristics on differential effectiveness were statistically underpowered. However, it is remarkable that the majority of these studies involved workers in lower socioeconomic position in the development and implementation of the programme. The citizen science approach, actively involving disadvantaged people in research, is increasingly considered as a promising way of promoting the health of disadvantaged groups and decreasing inequalities.^35^ By active involvement of workers in the development of WHPPs, the programmes are most likely better targeted to the needs and priorities of the target group.^36^

### Strengths and limitations

The strengths of this study are the systematic comparison of studies testing the differential effectiveness of WHPPs between low and high socioeconomic groups, and the comparison between qualitative information on differential effectiveness and quantitative information on programme effectiveness among workers in low socioeconomic position compared with programme effectiveness among those in higher socioeconomic groups.

To our knowledge this review is the first to systematically evaluate differences in effectiveness of the same WHPPs between low and high socioeconomic groups, including studies from different countries. Therefore, statements on differential effectiveness of WHPPs and types of interventions conducive to decreasing socioeconomic inequalities can be generalised to a larger population.

This study also has limitations. The first limitation concerns the search of eligible studies, which was initially done by searching through reviews published since 2013. The decision to perform this two-tier search was pragmatically based on the high number of studies published on WHPPs. Because we limited the comprehensiveness of our search by searching in reviews, we could have missed other eligible studies. However, the advantages of our two-tier strategy are that eligible studies were identified faster and more time was spent on screening and data extraction. In addition, a wide search can be imprecise and requires a lot of time spent on screening irrelevant studies.^37^

Another limitation is that the majority of the included studies were not primarily designed to test the differences in effectiveness of WHPPs. As a result these studies were not sufficiently powered to determine differential effects across socioeconomic groups. Although estimates of differential effectiveness in small studies may not be very precise and should be interpreted with caution, they are essential for providing policy information on the effects of interventions for different subgroups and how WHPPs may help to decrease socioeconomic inequalities in health.^38^ Therefore, future studies should be designed to test differential effectiveness between socioeconomic groups with a sufficient power.

Furthermore, the results of our study could be influenced by publication bias. Results indicating equal effectiveness of WHPPs between socioeconomic groups or higher effectiveness for workers in higher socioeconomic position might have been under-reported in the scientific literature, and therefore the results of this study could overestimate the potential of WHPPs to decrease socioeconomic inequalities in health.

Finally, a limitation is the large heterogeneity of the included studies with respect to design and presentation of results. The follow-up period to determine the effectiveness of WHPPs varied greatly, namely between 3 months and 3 years. In addition, the included studies used different effect measures to present the differential effectiveness of WHPPs between socioeconomic groups. This limits the comparability of the results.

## Conclusion

This systematic review does not indicate that WHPPs increase socioeconomic inequalities in health. The limited quantitative information available suggests that WHPPs may contribute to reducing socioeconomic inequalities. However, more insight into socioeconomic differences in effectiveness of WHPPs is needed to develop strategies to decrease socioeconomic health inequalities in the workforce.

## References

[1] World Health Organization Non communicable diseases country profiles 2018, 2018 Available: http://apps.who.int/iris/bitstream/handle/10665/274512/9789241514620-eng.pdf?ua=1 [Accessed Jan 2019].

[2] Gordois AL, Toth PP, Quek RG, et al Productivity losses associated with cardiovascular disease: a systematic review. Expert Rev Pharmacoecon Outcomes Res 2016;16:759–69. 10.1080/14737167.2016.1259571 27831848

[3] van Duijvenbode DC, Hoozemans MJM, van Poppel MNM, et al The relationship between overweight and obesity, and sick leave: a systematic review. Int J Obes 2009;33:807–16. 10.1038/ijo.2009.121 19528969

[4] Virtanen M, Ervasti J, Head J, et al Lifestyle factors and risk of sickness absence from work: a multicohort study. Lancet Public Health 2018;3:e545–54. 10.1016/S2468-2667(18)30201-9 30409406 PMC6220357

[5] Mackenbach JP, Stirbu I, Roskam A-JR, et al Socioeconomic inequalities in health in 22 European countries. N Engl J Med Overseas Ed 2008;358:2468–81. 10.1056/NEJMsa0707519 18525043

[6] Mackenbach JP, Valverde JR, Artnik B, et al Trends in health inequalities in 27 European countries. Proc Natl Acad Sci U S A 2018;115:6440–5. 10.1073/pnas.1800028115 29866829 PMC6016814

[7] Barsanti S, Salmi L-R, Bourgueil Y, et al Strategies and governance to reduce health inequalities: evidences from a cross-European survey. Glob Health Res Policy 2017;2:18 10.1186/s41256-017-0038-7 29202086 PMC5683456

[8] World Health Organization Regional office for Europe Good practice in occupational health services: a contribution to workplace health, 2002 Available: http://www.euro.who.int/document/e77650.pdf [Accessed Dec 2018].

[9] Stokols D, Pelletier KR, Fielding JE The ecology of work and health: research and policy directions for the promotion of employee health. Health Educ Q 1996;23:137–58. 10.1177/109019819602300202 8744869

[10] Tam G, Yeung MPS A systematic review of the long-term effectiveness of work-based lifestyle interventions to tackle overweight and obesity. Prev Med 2018;107:54–60. 10.1016/j.ypmed.2017.11.011 29155225

[11] Ananthapavan J, Peterson A, Sacks G Paying people to lose weight: the effectiveness of financial incentives provided by health insurers for the prevention and management of overweight and obesity - a systematic review. Obes Rev 2018;19:605–13. 10.1111/obr.12657 29266677

[12] Brinkley A, McDermott H, Munir F What benefits does team sport hold for the workplace? A systematic review. J Sports Sci 2017;35:136–48. 10.1080/02640414.2016.1158852 26979430

[13] Hendren S, Logomarsino J Impact of worksite cafeteria interventions on fruit and vegetable consumption in adults. Intl J of Workplace Health Mgt 2017;10:134–52. 10.1108/IJWHM-12-2016-0089

[14] Allan J, Querstret D, Banas K, et al Environmental interventions for altering eating behaviours of employees in the workplace: a systematic review. Obes Rev 2017;18:214–26. 10.1111/obr.12470 27860169

[15] Rongen A, Robroek SJW, van Lenthe FJ, et al Workplace health promotion: a meta-analysis of effectiveness. Am J Prev Med 2013;44:406–15. 10.1016/j.amepre.2012.12.007 23498108

[16] Cairns J-M, Bambra C, Hillier-Brown FC, et al Weighing up the evidence: a systematic review of the effectiveness of workplace interventions to tackle socio-economic inequalities in obesity. J Public Health 2015;37:659–70. 10.1093/pubmed/fdu077 PMC5896794 25316262

[17] Montano D, Hoven H, Siegrist J A meta-analysis of health effects of randomized controlled worksite interventions: does social stratification matter? Scand J Work Environ Health 2014;40:230–4. 10.5271/sjweh.3412 24788850

[18] Magnée T, Burdorf A, Brug J, et al Equity-specific effects of 26 Dutch obesity-related lifestyle interventions. Am J Prev Med 2013;44:e61–70. 10.1016/j.amepre.2012.11.041 23683991

[19] Higgins JPT, Green S Cochrane Handbook for systematic reviews of interventions, version 5.1.0. The Cochrane Collaboration, Wiley Online Library, 2011.

[20] Bergström G, Björklund C, Fried I, et al A comprehensive workplace intervention and its outcome with regard to lifestyle, health and sick leave: the AHA study. Work 2008;31:167–80. 18957735

[21] Sorensen G, Barbeau E, Stoddard AM, et al Promoting behavior change among working-class, multiethnic workers: results of the healthy directions--small business study. Am J Public Health 2005;95:1389–95. 10.2105/AJPH.2004.038745 16006422 PMC1449371

[22] Cook RF, Hersch RK, Schlossberg D, et al A web-based health promotion program for older workers: randomized controlled trial. J Med Internet Res 2015;17:e82 10.2196/jmir.3399 25830503 PMC4390614

[23] Robroek SJW, Polinder S, Bredt FJ, et al Cost-Effectiveness of a long-term internet-delivered worksite health promotion programme on physical activity and nutrition: a cluster randomized controlled trial. Health Educ Res 2012;27:399–410. 10.1093/her/cys015 22350194 PMC3337425

[24] Slootmaker SM, Chinapaw MJM, Schuit AJ, et al Feasibility and effectiveness of online physical activity advice based on a personal activity monitor: randomized controlled trial. J Med Internet Res 2009;11:e27 10.2196/jmir.1139 19674956 PMC2763404

[25] Reijonsaari K, Vehtari A, Kahilakoski O-P, et al The effectiveness of physical activity monitoring and distance counseling in an occupational setting - results from a randomized controlled trial (CoAct). BMC Public Health 2012;12:344 10.1186/1471-2458-12-344 22578104 PMC3507818

[26] Bhiri S, Maatoug J, Zammit N, et al A 3-year workplace-based intervention program to control noncommunicable disease risk factors in Sousse, Tunisia. J Occup Environ Med 2015;57:e72–7. 10.1097/JOM.0000000000000500 26147554

[27] Sorensen G, Stoddard A, Hunt MK, et al The effects of a health promotion-health protection intervention on behavior change: the WellWorks study. Am J Public Health 1998;88:1685–90. 10.2105/AJPH.88.11.1685 9807537 PMC1508574

[28] Sorensen G, Stoddard AM, LaMontagne AD, et al A comprehensive worksite cancer prevention intervention: behavior change results from a randomized controlled trial (United States). Cancer Causes Control 2002;13:493–502. 10.1023/A:1016385001695 12195637

[29] van den Brand FA, Nagelhout GE, Winkens B, et al Effect of a workplace-based group training programme combined with financial incentives on smoking cessation: a cluster-randomised controlled trial. Lancet Public Health 2018;3:e536–44. 10.1016/S2468-2667(18)30185-3 30342875

[30] Sorensen G, Pednekar M, Cordeira LS, et al Effects of a worksite tobacco control intervention in India: the Mumbai worksite tobacco control study, a cluster-randomised trial. Tob Control 2017;26:210–6. 10.1136/tobaccocontrol-2015-052671 26883793 PMC4987266

[31] Scoggins JF, Sakumoto KN, Schaefer KS, et al Short-Term and long-term weight management results of a large employer-sponsored wellness program. J Occup Environ Med 2011;53:1215–20. 10.1097/JOM.0b013e3182338676 22068128

[32] Carpenter KM, Vickerman KA, Salmon EE, et al A randomized pilot study of a Phone-Based mindfulness and weight loss program. Behav Med 2019;45:1–11. 10.1080/08964289.2017.1384359 28985151 PMC8462129

[33] Tudor Hart J, Hart JT The inverse care law. The Lancet 1971;297:405–12. 10.1016/S0140-6736(71)92410-X 4100731

[34] Lorenc T, Petticrew M, Welch V, et al What types of interventions generate inequalities? Evidence from systematic reviews. J Epidemiol Community Health 2013;67:190–3. 10.1136/jech-2012-201257 22875078

[35] Den Broeder L, Lemmens L, Uysal S, et al Public health citizen science; perceived impacts on citizen scientists: a case study in a low-income neighbourhood in the Netherlands. Citiz Sci 2017;21:7201–17.

[36] Hancock L, Sanson-Fisher RW, Redman S, et al Community action for health promotion: a review of methods and outcomes 1990-1995. Am J Prev Med 1997;13:229–39. 10.1016/S0749-3797(18)30168-5 9236957

[37] Ogilvie D, Hamilton V, Egan M, et al Systematic reviews of health effects of social interventions: 1. finding the evidence: how far should you go? J Epidemiol Community Health 2005;59:804–8. 10.1136/jech.2005.034181 16100321 PMC1733146

[38] Petticrew M, Tugwell P, Kristjansson E, et al Damned if you do, damned if you don't: subgroup analysis and equity. J Epidemiol Community Health 2012;66:95–8. 10.1136/jech.2010.121095 21652518

