# Supplementary material for: Are workplace health promotion programmes effective for all socioeconomic groups? A systematic review
Source: Occup Environ Med. 2020 Mar 26;77(9):589–96. doi: 10.1136/oemed-2019-106311 (PMC7547856; doi:10.1136/oemed-2019-106311)
Supplement: Supplementary data [file oemed-2019-106311supp001.pdf]

Are workplace health promotion programmes effective for all socioeconomic groups? A systematic review

David van de Ven[1], Suzan J W Robroek[1], Alex Burdorf[1]

[1] Department of Public Health, Erasmus MC, University Medical Center Rotterdam, Rotterdam, The Netherlands

Appendix A. Search strategy

Embase

('lifestyle'/exp OR 'lifestyle modification'/exp OR 'sedentary lifestyle'/exp OR 'physical activity'/exp OR 'sitting'/exp OR exercise/exp OR sport/exp OR 'health behavior'/de OR 'alcohol consumption'/de OR 'substance abuse'/exp OR 'drinking behavior'/exp OR 'smoking cessation'/exp OR smoking/exp OR 'smoking ban'/de OR obesity/exp OR 'body mass'/exp OR 'body weight'/de OR 'body weight change'/exp OR 'body weight control'/exp OR 'body weight gain'/exp OR 'body weight loss'/exp OR 'waist circumference'/de OR 'waist hip ratio'/de OR 'skinfold thickness'/de OR 'body fat'/de OR 'body composition'/de OR 'body distribution'/de OR 'body fat distribution'/de de OR 'dietary intake'/exp OR 'healthy diet'/de OR 'unhealthy diet'/de OR 'dietary compliance'/de OR nutrition/de OR 'tobacco smoke'/de OR 'behavior change'/de OR diet/de OR 'feeding behavior'/exp OR 'motor activity'/de OR alcoholism/exp OR (lifestyle OR 'life style' OR tobacco OR smoking OR smoker* OR cigarette* OR alcohol* OR addict* OR drinking OR sedentar* OR (physical* NEAR/3 (activ* OR inactiv*)) OR exercis* OR walking OR cycling OR jogging OR sitting OR running OR sport* OR obes* OR adiposit* OR overweight* OR (body NEAR/3 (mass OR weight OR fat OR composition* OR distribut*)) OR (weight NEAR/3 (gain OR change OR loss OR reduc*)) OR overweight OR bmi OR (waist NEAR/3 (circumferen* OR hip)) OR (skinfold* NEAR/3 thick*) OR diet* OR ((eating OR feeding OR calor* OR fat OR energ* OR salt OR natrium) NEAR/3 (intake* OR behav* OR restrict* OR health* OR unhealth* OR intake*)) OR ((health* OR chang*) NEAR/3 behav*) OR 'fast food' OR fruit OR vegetable* OR nutrition* OR (active* NEAR/3 commut*)):ab,ti,kw) AND ('intervention study'/exp OR 'program evaluation'/exp OR 'education program'/exp OR 'smoking cessation program'/exp OR 'health education'/exp OR 'clinical trial (topic)'/de OR (intervention* OR program* OR (health NEAR/3 (education* OR promot*)) OR effectiv* OR evaluation* OR trial* OR campaign*):ab,ti,kw) AND ('workplace'/exp OR Employee/de OR employer/de OR worker/de OR 'white collar worker'/de OR 'blue collar worker'/de OR 'work environment'/de OR (worker OR workers OR workplace* OR worksite* OR workstation* OR work-based OR workbased OR employee* OR employer* OR (work* NEAR/3 population*) OR 'at work' OR workforce* OR work-force*):ab,ti,kw) AND ('systematic review'/exp OR 'meta analysis'/de OR ((systematic* NEAR/3 review*) OR meta-analy*):ab,ti) AND [english]/lim NOT (([Conference Abstract]/lim AND [1800-2016]/py) OR [Letter]/lim OR [Note]/lim OR [Editorial]/lim)

Medline Ovid

(exp life style/ OR exp Motor Activity/ OR exp sports/ OR exp health behavior/ OR exp drinking behavior/ OR exp "Tobacco Use"/ OR exp obesity/ OR Body Mass Index/ OR exp Diet/ OR feeding behavior/ OR sports/ OR exp Running/ OR Bicycling/ OR body weight/ OR exp body weight changes/ OR Waist Circumference/ OR Waist-Hip Ratio/ OR skinfold thickness/ OR exp body composition/ OR Energy

Intake/ OR healthy diet/ OR exp Substance-Related Disorders/ OR exp smoking/ OR Smoke-Free Policy/ OR exp Motor Activity/ OR exp Alcoholism/ OR (lifestyle OR life style OR tobacco OR smoking OR smoker* OR cigarette* OR alcohol* OR addict* OR drinking OR sedentar* OR (physical* ADJ3 (activ* OR inactiv*)) OR exercis* OR walking OR cycling OR jogging OR sitting OR running OR sport* OR obes* OR adiposit* OR overweight* OR (body ADJ3 (mass OR weight OR fat OR composition* OR distribut*)) OR (weight ADJ3 (gain OR change OR loss OR reduc*)) OR overweight OR bmi OR (waist ADJ3 (circumferen* OR hip)) OR (skinfold* ADJ3 thick*) OR diet* OR ((eating OR feeding OR calor* OR fat OR energ* OR salt OR natrium) ADJ3 (intake* OR behav* OR restrict* OR health* OR unhealth* OR intake*)) OR ((health* OR chang*) ADJ3 behav*) OR fast food OR fruit OR vegetable* OR nutrition* OR (active* ADJ3 commut*)).ab,ti,kw.) AND (exp program evaluation/ OR education/ OR education.xs. OR exp health education/ OR exp Clinical Trials as Topic/ OR (intervention* OR program* OR (health ADJ3 (education* OR promot*)) OR effectiv* OR evaluation* OR trial* OR campaign*).ab,ti,kw.) AND (workplace/ OR (worker OR workers OR workplace* OR worksite* OR workstation* OR work-based OR workbased OR employee* OR employer* OR (work* ADJ3 population*) OR at work OR workforce* OR work-force*).ab,ti,kw.) AND (Meta-Analysis/ OR ((systematic* ADJ3 review*) OR meta-analy*).ab,ti.)

Cochrane DSR DARE

((lifestyle OR 'life style' OR tobacco OR smoking OR smoker* OR cigarette* OR alcohol* OR addict* OR drinking OR sedentar* OR (physical* NEAR/3 (activ* OR inactiv*)) OR exercis* OR walking OR cycling OR jogging OR sitting OR running OR sport* OR obes* OR adiposit* OR overweight* OR (body NEAR/3 (mass OR weight OR fat OR composition* OR distribut*)) OR (weight NEAR/3 (gain OR change OR loss OR reduc*)) OR overweight OR bmi OR (waist NEAR/3 (circumferen* OR hip)) OR (skinfold* NEAR/3 thick*) OR diet* OR ((eating OR feeding OR calor* OR fat OR energ* OR salt OR natrium) NEAR/3 (intake* OR behav* OR restrict* OR health* OR unhealth* OR intake*)) OR ((health* OR chang*) NEAR/3 behav*) OR 'fast food' OR fruit OR vegetable* OR nutrition* OR (active* NEAR/3 commut*)):ab,ti,kw) AND ((intervention* OR program* OR (health NEAR/3 (education* OR promot*)) OR effectiv* OR evaluation* OR trial* OR campaign*):ab,ti,kw) AND ((worker OR workers OR workplace* OR worksite* OR workstation* OR work-based OR workbased OR employee* OR employer* OR (work* NEAR/3 population*) OR 'at work' OR workforce* OR work-force*):ab,ti,kw)

Appendix B. Methodological quality criteria

Criteria	Definition
**A. Randomization procedure**	Positive if there was a clear description of the randomization procedure and if the randomization was adequately performed (i.e., by a random select of numbers or by a computer-generated list)
**B. Similarity of groups**	Positive if the study groups were similar at the beginning of the study with regard to the relevant outcome variable (p<0.05)
**C. Blinding of participants**	Positive if the participant was unaware of being assigned to the intervention group or control group
**D. Compliance**	Positive if participants attended the intervention satisfactorily according to the opinion of the reviewers
**E. Loss to follow-up**	Positive if the percentage of drop-outs during the study period did not exceed 20% for short-term follow-up (≤3 months) or 30% for long-term follow-up (>3 months)
**F. Intention-to-treat**	Positive if an intention-to-treat analysis was performed for the outcome variable
**G. Controlled for confounders**	Positive if the analysis was controlled for potential confounders
**H. Data-collection method**	Positive if objective measures were used for data collection on the outcome variable(s)
**I. Follow-up**	Positive if follow-up was ≥6 months

Appendix C. Flowcharts literature search

Search through systematic reviews/meta-analyses

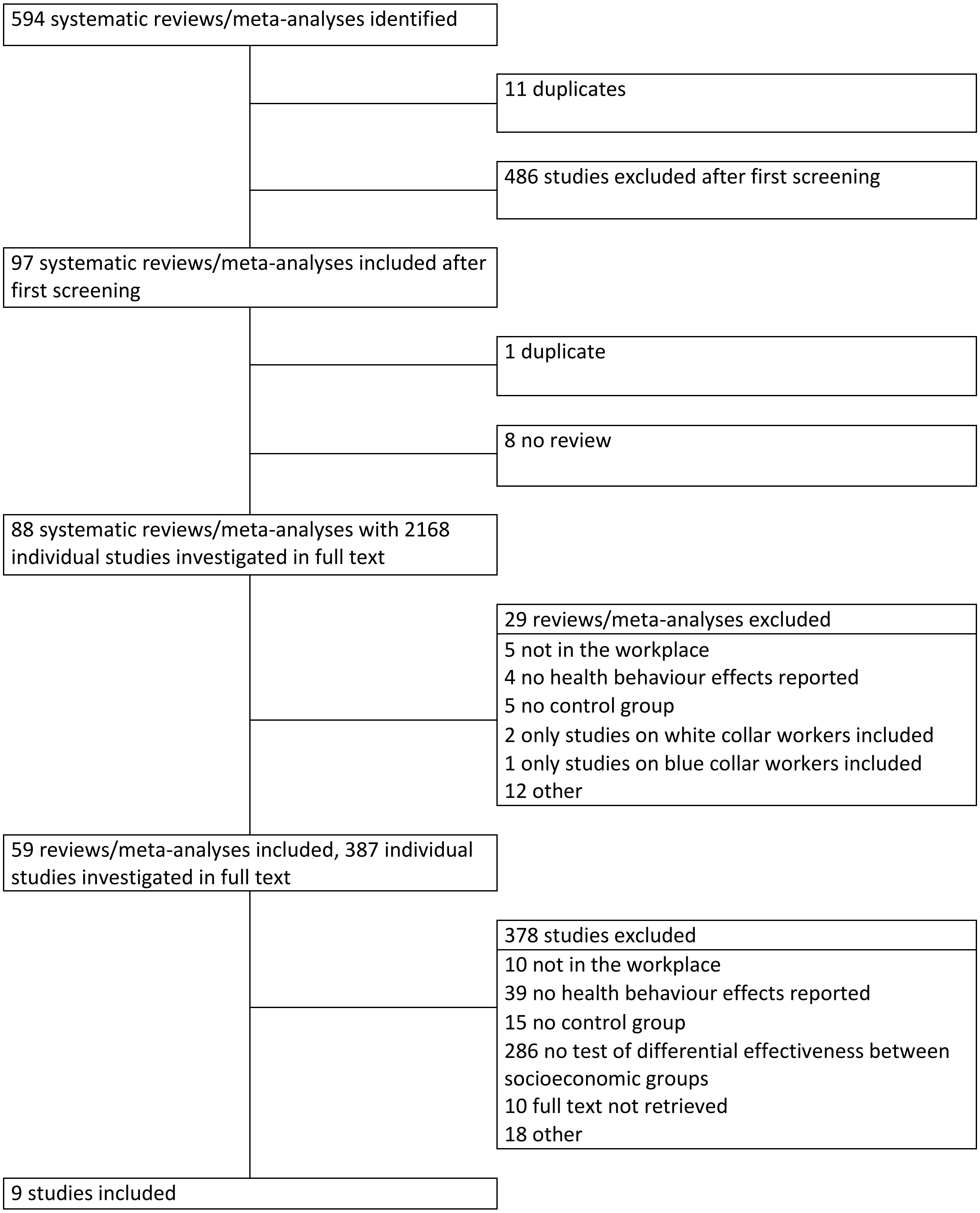

Additional search

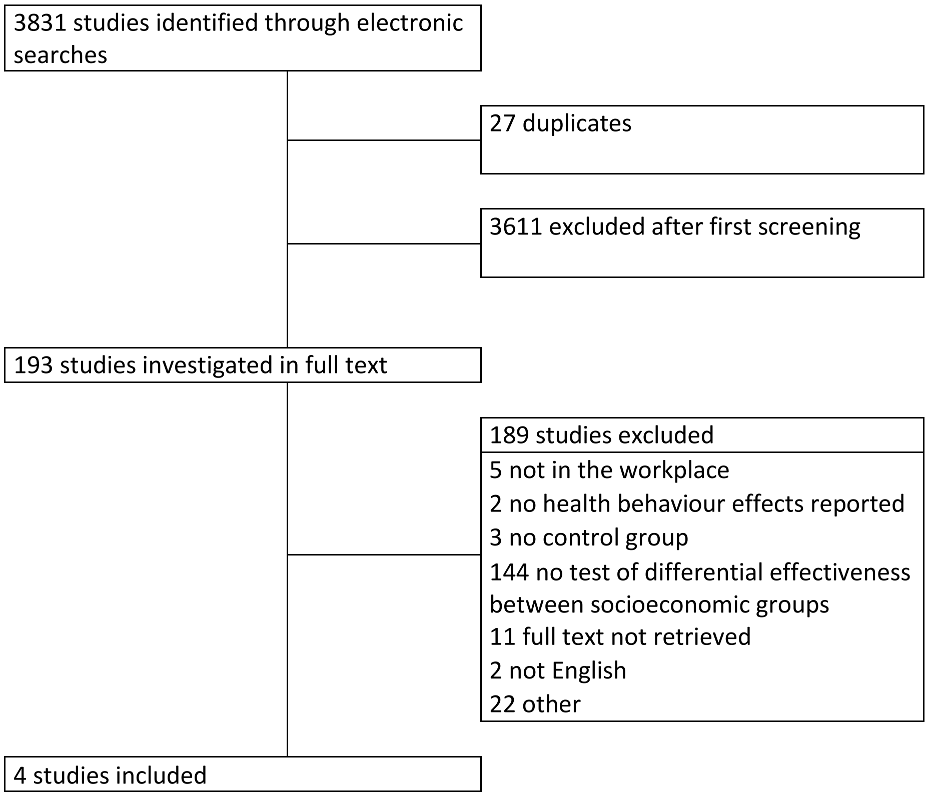

Appendix D. Programme content, study- and population characteristics of included WHPPs

Study	Country	Programme content	Health behaviour (s)	Design	Follow-up period	N baseline	Measure of socioeconomic position	Proportion of workers with low SEP	Type of industry
Bergstrom et al. (2008)[20]	Sweden	Screening (health, lifestyle, work-related factors), feedback, individually directed interventions	Smoking and physical activity	CT	42 months	2894	Occupational status (blue- versus white-collar)	80% blue-collar workers (vs. white-collars workers)	Processing and engineering (paper mills, steel works, truck manufacturers)
Sorensen et al. (2005)[21]	United States	Participatory activities (i.e. group discussions, demonstrations), worksite-wide events (i.e. health fairs), individually tailored interventions focused on risk factors, and environmental changes (healthy eating, tobacco control, reduction of hazardous occupational exposures.	Nutrition and physical activity	Cluster -RCT	18 months	1740	Occupational class (managers vs. workers), education (high school, post-high school, college degree or more), and household income	83.5% workers (vs. managers)	Manufacturing (products and services for the automobile industry, electronics, and other businesses)

Sorensen et al. (1998)[27]	United States	Worksite environmental changes (i.e. tobacco control policies, increased availability of healthy foods, and reduction in the potential for exposure to occupational hazards), and health education programs targeting individual behaviours in each of the risk factor areas.	Nutrition and smoking	Cluster -RCT	2 years	5914	Job category (skilled/unskilled, office work, professional/managerial/administrative)	43% skilled and unskilled labourers (vs. office workers, and professionals/managers)	Manufacturing (industrial, chemical, and other products)
Sorensen et al. (2002)[28]	United States	Worksite environmental changes (i.e. tobacco control policies, healthy eating, reduction of workers' exposure to hazardous substances), individual interventions targeting occupational health and safety, and health behaviour (i.e. self-assessment with feedback, self-help activities,	Smoking and nutrition	Cluster -RCT	24 months	9019	Job type (Hourly workers vs. salaried workers)	68.2% hourly workers (vs. salaried workers)	Manufacturing (chemicals, food, technology, automobile parts, metal, paper, other)

		contests, demonstrations or displays with personal interactions, opportunities to try behaviours and set goals, and group discussion)							
Cook et al. (2015)[22]	United States	A web-based multimedia program containing information and guidance on risk factors (diet, physical activity, stress, tobacco use), assessments across major health topics.	Nutrition, BMI, and physical activity	RCT	3 months	278	Education (High school-graduate), income	25.8% low education (vs high education)	Information technology
Robroek et al. (2012)[23]	The Netherlands	Computer-tailored advice (including personal and action feedback, taking into account perceived barriers for participants not meeting the guidelines,) on physical activity and nutrition, online self-monitors, a food frequency questionnaire, possibility to submit	Physical activity, nutrition, and BMI	Cluster-RCT	24 months	924	Education; low (primary school, lower and intermediate secondary schooling or lower vocational training), intermediate (higher secondary schooling or intermediate	22.1% low education (vs. Intermediate and high education)	Various (health care, commercial services, executive branch of government)

		particular questions to several health professionals.					vocational schooling) and high (higher vocational schooling or university)		
Slootmaker et al. (2009)[24]	The Netherlands	Receipt of advice to increase PA levels; personal activity monitor (PAM) and concise web-based tailored PA advice	Physical activity and body weight	RCT	8 months	102	Education; low vs. High (higher vocational education or a university degree)	35% low education (vs. high education)	Office work (not specified)
Scoggins et al. (2011)[31]	United States	HRA, individual action plans, programs and tools emphasizing healthy eating, stress management, and weight management, environmental changes (healthy food options and portions, prompts to use the stairs)	BMI	Prospective cohort	1 year	19559	Education (college graduate, some college, high school only, no high school)	12.6% no high school and high school only (some college and college graduate)	Governmental
Reijonsaari et al. (2012)[25]	Finland	Written results of physical exams, general information on physical activity and health, activity monitoring and distance counselling	Physical activity	RCT	1 year	544	Occupational status; clerical employees versus experts/managers	89.6% clerical (vs experts/managers)	Insurance

Bhiri et al. (2015)[26]	Tunisia	Health education programs (educative films, comprehensive educative sessions, workshops about healthy diet and tobacco cessation, open sensitization days focusing on the three main NCD risk factors), environmental activities (physical activity facilities at the workplaces, banning smoking at the workplaces.)	Nutrition, physical activity and smoking	CT	3 years	1775	Educational level (low, medium, high), SEL (low, medium, high), Job type (worker, office staff)	62% low SEL (vs medium and high SEL)	Various (not specified)
Carpenter et al. (2017)[32]	United states	Proactive coaching calls. Call topics included mindfulness meditation, mindfulness of everyday activities, mindful eating, acceptance of thoughts and emotions, and self-compassion. In addition, participants were emailed links to brief educational e-lessons, which included links to resources and	Body weight	RCT	6 months	75	Education (High school/GED or less, Some college, College)	5.3% high school/GED or less (vs. some college and college)	Various (not specified)

		downloadable mindfulness exercises.							
Van den Brand et al. (2018)[29]	The Netherl ands	Smoking cessation group training and vouchers for being abstinent (€50 at the end of the training program, €50 3 months after completion of the program, €50 after 6 months, and €200 after 12 months).	Smoking	Cluster -RCT	12 months	604	Education; low (none completed, primary school and lower secondary education), moderate (middle secondary education) and high (upper secondary education and university) and net monthly household income	29,6% low education (vs moderate and high education)	Various (not specified)
Sorensen et al. (2017)[30]	India	Tobacco health education events (worker level), program planning meetings, occupational safety and health consultation, tobacco policy consultation (management level)	Smoking	Cluster -RCT	14-18 months	6880	Production versus non-production (managers and office workers) based on job title	76.5% production workers (vs. managers and office workers)	Manufacturing (food, chemical products, steel, industrial machinery, electricity, metal, petroleum, printing)

Appendix E. Methodological quality rating of included studies

Criteria	A. Randomization procedure	B. Similarity of the groups	C. Blinding of participants	D. Compliance	E. Loss to follow up	F. Intention-to-treat	G. Controlled for confounders	H. Data-collection method	I. Follow-up	Total score
**Author**										
Bergström et al. (2008)[20]	0	0	0	1	0	1	0	0	1	3
Sorensen et al. (2005)[21]	1	1	0	1	0	1	1	0	1	6
Sorensen et al. (1998)[27]	1	1	0	0	0	1	1	0	1	5
Sorensen et al. (2002)[28]	1	1	0	0	0	1	1	0	1	5
Cook et al. (2015)[22]	1	1	0	1	1	1	1	0	0	6
Robroek et al. (2012)[23]	1	1	1	0	0	1	1	0.5	1	6.5
Slootmaker et al. (2009)[24]	1	1	0	1	1	1	1	0.5	1	7.5
Scoggins et al. (2011)[31]	0	1	0	0	1	1	1	0	1	5
Reijonsaari et al. (2012)[25]	1	1	0	0.5	0	1	1	0	1	5.5
Bhiri et al. (2015)[26]	0	0.5	0	0	0	1	0	0	1	2.5
Carpenter et al. (2017)[32]	1	1	0	0.5	1	1	1	1	1	7.5
Van den Brand et al. (2018)[29]	1	1	0	1	1	1	1	1	1	8
Sorensen et al. (2017)[30]	1	1	0	0	1	1	1	0	1	6

Appendix F. Number of studies and comparisons reporting on the presence or absence of differential effectiveness and (pooled) effectiveness ratios by program characteristic

	Equally effective	More effective for lower SEP
Program component		
Direct coaching (n=7, k=15)	(n=5, k=11)[20 25 28 29 32]	n/a
	Pooled ratio=2.53, 2.46:2.80 (k=2)[28 30]	Pooled ratio=3.24, 1.65:3.36 (k=2)[30 31]
Education (n=10, k=64)	(n=7, k=43)[21-24 27 28 32]	(n=1, k=3)[24]
	Pooled ratio=1.35, 0.54:3.04 (k=13)[21 26-28 30]	Pooled ratio=2.88, 1.65:3.36 (k=5)[21 27 30 31]
Environmental (n=5, k=25)	(n=2, k=8)[21 27]	n/a
	Pooled ratio=1.32, 0.54:3.04 (k=12)[21 26 27 30]	Pooled ratio=2.88, 1.65:3.36 (k=5)[21 27 30 31]
Financial incentive (n=1, k=1)	(n=1, k=1)[29]	
	n/a	
Population of workers		
Selective (n=3, k=21)	(n=3, k=18)[24 29 32]	(n=1, k=3)[24]
	n/a	n/a
Universal (n=10, k=54)	(n=7, k=36)[20-23 25 27 28]	n/a
	Pooled ratio=1.35, 0.54:3.04 (k=13)[20 21 26-28 30]	Pooled ratio=2.88, 1.65:3.36 (k=5)[21 27 30 31]
Involvement of workers in low socioeconomic position		
Yes (n=5, k=19)	(n=5, k=11)[20 21 27 28 30]	n/a
	Pooled ratio=2.47, 2.19:3.04 (k=4)[21 27 28 30]	Pooled ratio=2.90, 2.80:3.36 (k=4)[21 27 30]
No (n=8, k=56)	(n=7, k=43)[22-26 29 32]	(n=1, k=3)[24]
	Pooled ratio=0.91, 0.54:1.96 (k=9)[26]	Ratio 1.65 (k=1)[31]
Designed for workers in low socioeconomic position		
Yes (n=4, k=17)	(n=3, k=9)[21 27 28]	n/a
	Pooled ratio=2.47, 2.19:3.04 (k=4)[21 27 28 30]	Pooled ratio=2.90, 2.80:3.36 (k=4)[21 27 30]
No (n=9, k=58)	(n=7, k=45)[20 22-25 29 32]	(n=1, k=3)[24]
	Pooled ratio=0.91, 0.54:1.96 (k=9)[26]	Ratio 1.65 (k=1)[31]

None of the included studies reported or showed lower programme effectiveness for workers in low socioeconomic position
The sum of studies is higher than 13 because some studies test multiple comparisons of differential effectiveness with different results
The comparisons for which the (pooled) ratios could be calculated are distinguished from the comparisons for which only qualitative information was available
n, number of studies; k, number of comparisons.
